# LncZEB1-AS1 regulates hepatocellular carcinoma bone metastasis via regulation of the miR-302b-EGFR-PI3K-AKT axis

**DOI:** 10.7150/jca.45995

**Published:** 2020-06-28

**Authors:** Zhen-jiang Ma, Yao Wang, Hui-fen Li, Ming-Hua Liu, Feng-rui Bi, Long Ma, Hui Ma, Hong-li Yan

**Affiliations:** 1 Department of Orthopedics, the Third Affiliated Hospital of Second Military Medical University, Shanghai 201805, P.R. China.; 2 Department of Orthopedics, Shanghai Ninth People's Hospital, Shanghai 200011, P.R. China.; 3 Department of Laboratory Medicine, Changhai Hospital, Second Military Medical University, Shanghai, P.R. China.; 4 Department of Laboratory Medicine, The Affiliated Wuxi Maternity and Child Health Care Hospital of Nanjing Medical University, Wuxi, Jiangsu 214000, China.; 5 Department of Interventional, Eastern Hepatobiliary Surgery Hospital, Second Military Medical University, Shanghai, P.R. China.

**Keywords:** Hepatocellular carcinoma, Bone metastasis, LncZEB1-AS1, miR-302b, EGFR-PI3K-AKT axis

## Abstract

In patients with hepatocellular carcinoma (HCC), disease progression and associated bone metastasis (BM) can markedly reduce quality of life. While the long non-coding RNA (lncRNA) zinc finger E-box binding homeobox 1 antisense 1 (ZEB1-AS1) has been shown to function as a key regulator of oncogenic processes in HCC and other tumor types, whether it plays a role in controlling HCC BM remains to be established. In the current study, we detected the significant upregulation of lncZEB1-AS1 in HCC tissues, and we found this expression to be associated with BM progression. When we knocked down this lncRNA in HCC cells, we found that this significantly reduced their migratory, invasive, and metastatic activity both *in vitro* and *in vivo.* At a mechanistic level, we found that lncZEB1-AS1 was able to target miR-302b and to thereby increase PI3K-AKT pathway activation and EGFR expression, resulting in the enhanced expression of downstream matrix metalloproteinase genes in HCC cells. In summary, our results provide novel evidence that lncZEB1-AS1 can promote HCC BM through a mechanism dependent upon the activation of PI3K-AKT signaling, thus highlighting a potentially novel therapeutic avenue for the treatment of such metastatic progression in HCC patients.

## Introduction

Hepatocellular cancer (HCC) accounts for up to 90% of all liver cancer cases and is particularly prevalent in regions including sub-Saharan Africa and Eastern Asia 1, 2. At present, HCC remains the sixth most common and third deadliest cancer type, with over 780,000 new cases and almost 750,000 deaths in 2012 alone 2. Several major advances in recent years have helped to prolong HCC patient survival 3, but tumor metastasis is still associated with poor patient outcomes and limited long-term survival in those with 4. Bone metastasis (BM) in particular is detected in roughly 38.5% of HCC patients exhibiting extrahepatic metastases 5, 6 and in 11.7% of HCC patients undergoing curative respective surgery 7. BM is a serious HCC complication, as it is associated with the potential for bone fractures, severe pain, nerve compression 8, and a median survival of just 7.4 months 9. Therefore, investigating the molecular mechanisms HCC BM is important will improve the diagnosis and treatment of this condition.

Long noncoding RNAs (lncRNAs) are RNAs which are >200 nucleotides long and which generally lack the potential to code for protein 10. Dysregulated lncRNA expression profiles have been shown to be associated with a range of pathological processes including aberrant proliferation 11, angiogenesis 12, and tumor metastasis 13. How lncRNAs regulate BM in HCC patients, however, is not well understood 14, 15. Most lncRNAs are found within cellular nuclei 16, wherein they regulate a number of biological activities at the transcriptional and posttranscriptional levels 17. The zinc finger E-box binding homeobox 1 antisense 1 (ZEB1-AS1) lncRNA which is encoded in the 10p11.22 region contiguous with ZEB1 has been well-characterized as an oncogenic lncRNA 18. ZEB1 is a key transcription factor associated with tumor cell metastasis, with lncZEB1-AS1 being an antisense transcript derived from the ZEB1 promoter 19. Early studies found that lncZEB1-AS1 overexpression was evident in the context of esophageal squamous cell carcinoma 20, while more recent studies have confirmed that it is also dysregulated in colorectal cancer, gastric cancer, and HCC 18, 21, 22. In gastric cancer patients, lncZEB1-AS1 overexpression has been found to be independently associated with reduced patient survival 23, whereas in colorectal cancer it is associated with enhanced tumor cell proliferation and migration through its regulation of the miR-101/ZEB1 axis 21. Recent work by Li *et al.* 18 found that increased lncZEB1-AS1 expression was evident in HCC tumor tissue samples relative to adjacent normal tissues (ANTs) observed in HCC tissues, compared with the adjacent normal tissues, and they further demonstrated that this lncRNA was able to modulate HCC cellular proliferation, migration, invasion, and cell cycle progression. In a separate study, lncZEB1-AS1 was suggested to regulate the progression of liver cancer owing to its ability to target miR-365a-3p 24. Whether lncZEB1-AS1 similarly regulates HCC BM development or progression, however, remains to be determined.

In this study, we determined that lncZEB1-AS1 is able to promote HCC BM via the epigenetic suppression of miR-302b expression, resulting in enhanced EGFR/PI3K-AKT signaling. Together, our results suggest that this signaling axis may be a viable diagnostic and/or therapeutic target for clinicians seeking to prevent HCC BM development.

## Material and Methods

### Patient samples

We collected HCC and paired ANT samples from 90 patients with HCC undergoing respective surgery or tissue biopsy. These patients had not undergone any targeted therapeutic treatments prior to sample collection. Collected samples were snap-frozen and stored at -80 °C. The research ethics committee of Eastern Hepatobiliary Surgery Hospital, Second Military Medical University approved this study, which was consistent with the Declaration of Helsinki. Patient characteristics and clinical findings are detailed in **Table 1.**

### Cell culture

Human HCC (PLC, MHCC-97H, Hep3B, and Huh7) and control (NCM-460) cell lines were from the Chinese Academy of Sciences (Shanghai, China), and were cultured in DMEM (Gibco, NY, USA) containing 10% FBS (Gibco) and penicillin/streptomycin (Sigma, MO, USA) at 37 °C in a humidified 5% CO2 incubator. PI3K activity was assessed as in a prior study 25.

### Transfection

LncZEB1-AS1-specific siRNA constructs (si-ZEB1-AS1#1, #2) and non-targeting control constructs (si-NC) were from Guangzhou RIBOBIO Co., Ltd (Guangzhou, China), while agomir/antagomir constructs specific for miR-302b and corresponding negative control constructs were from GenePharma Co., Ltd (Shanghai, China), as were the EGFR overexpression pcDNA3.1- EGFR (pc-EGFR) and empty control vectors. All cells were plated in 6-well plates for 24 h, followed by Lipofectamine 2000 (Invitrogen, USA)-mediated transfection.

### qRT-PCR

Total RNA was isolated from cells with the TRIzol reagent (Invitrogen), and RNA quantity and quality were then analyzed via a NanoDrop device (NanoDrop Technologies, USA). The expression of miR-302b was assessed using a Mir-X™ miRNA First-Strand Synthesis Kit (TaKaRa Biotechnology, Dalian, China) and a Mir-X™ miRNA qRT-PCR TB Green® Kit (TaKaRa), while lncZEB1-AS1 and EGFR expression were analyzed using a PrimeScript™ RT Reagent Kit (TaKaRa) and a SYBR-Green PCR Master Mix (TaKaRa). Relative mRNA and miRNA expression levels were quantified according to the 2-ΔΔCq method, with actin and U6 being used as normalization controls, respectively. Primers were as follows: ZEB1-AS1-Forward: 5'- CCGTGGGCACTGCTGAAT-3', ZEB1-AS1-Reverse: 5'- CTGCTGGCAAGCGGAACT-3'; MMP2-Forward: 5' - TGACTTTCTTGGATCGGGTCG-3', MMP2-Reverse: 5'-AAGCACCACATCAGATGACTG-3'; MMP7-Forward: 5'-GGTCACCTACAGGATCGTATCATAT-3', MMP7-Reverse: 5'-CATCACTGCATTAGGATCAGAGGAA-3'; MMP9-Forward: 5'-TGTACCGCTATGGTTACACTCG-3', MMP9-Reverse: 5'-GGCAGGGACAGTTGCTTCT-3'; E-cadherin -Forward: 5'-TACACTGCCCAGGAGCCAGA-3', E-cadherin -Reverse: 5'-TGGCACCAGTGTCCGGATTA-3'; N-cadherin -Forward: 5'-TCAGGCGTCTGTAGAGGCTT-3', N-cadherin-Reverse: 5'-ATGCACATCCTTCGATAAGACTG-3'; Vimentin-Forward: 5'-GACGCCATCAACACCGAGTT-3', Vimentin-Reverse: 5'-CTTTGTCGTTGGTTAGCTGGT-3'; miR-302b-Forward: 5'-ATCCAGTGCGTGTCGTG-3', miR-302b-Reverse: 5'-TGCTTAAGTGCTTCCATGTT-3'; ACTIN-Forward: 5'-GGGAAATCGTGCGTGACATTAAG-3', ACTIN-Reverse: 5'-GTGTTGGCGTACAGGTCTTTG-3'; U6-Forward: 5'-CTCGCTTCGGCAGCACA-3', U6-Reverse: 5'-AACGCTTCACGAATTTGCGT-3'.

### CCK-8 assay

After transfection, cells were incubated for 24 h prior to re-platting in 96-well plates (2×10^3^/well). Cells were then incubated for the indicated periods of time, after which each well was treated using 10 μL of CCK-8 solution (Shanghai Haling Biotechnology, Co., Ltd., Shanghai, China) at 37 °C for 2 h. Absorbance at 450 nm was then assessed via microplate reader (Bio-Rad, CA, USA).

### Transwell assays

Cells were allowed to rest for 48 h following transfection, after which 5×10^4^ cells were added to the upper chamber of an 8-µm pore size Transwell assay system (Corning, NY, USA) in 200 µL of serum-free media. For invasion assays, the membrane of this chamber was coated using Matrigel (BD Biosciences, NJ, USA), while for migration assays it was not. A 600 µL aliquot of FBS-containing media (10%) was added into the lower chamber for all assays, and cells were then incubated for 1 day. Non-migratory/invasive cells were gently removed with a swab, after which all remaining cells were methanol-fixed and stained with crystal violet. The cells in five random fields of view were then enumerated via microscopic analysis (Olympus Corporation, Tokyo, Japan).

### Bioinformatics analysis

StarBase 3.0 (http://starbase.sysu.edu.cn/) was used to predict potential binding targets for lncZEB1-AS1.

### RNA immunoprecipitation (RIP)

A Magna RIP RNA-Binding Protein Immunoprecipitation Kit (Millipore, MA, USA) was used based on provided directions in order to assess interactions between miR-302b and lncZEB1-AS1. Briefly, cellular lysates were mixed at 4 °C overnight with magnetic beads coated with a human AGO2 antibody or control IgG (Millipore). These beads were then collected, and qRT-PCR was used in order to analyze RNA enrichment in the precipitated fraction.

### Luciferase reporter assay

We obtained lncZEB1-AS1 constructs containing wither wild-type (WT) or mutated (MUT) versions of the putative miR-302b binding site from GenePharma, and we then inserted these fragments into the pmirGLO Dual-luciferase Vector (Promega, WI, USA) in order to produce the WT- and MUT-ZEB1-AS1 reporter constructs. This same strategy was used for EGFR reporter plasmid preparation. Prior to transfection, cells were added to 24-well plates overnight and were allowed to grow until reaching 60-70% confluence. Cells were then co-transfected with reporter plasmids along with either agomir-302b or agomir-NC. Following an additional 48 h incubation, the Dual-Luciferase Reporter Assay System (Promega) was utilized according to provided instructions, with Renilla luciferase activity being used as a normalization control.

### Western blotting

Total cellular protein was isolated with RIPA buffer (Beyotime Institute of Biotechnology), and a BCA assay kit (Beyotime Institute of Biotechnology) was then utilized to quantify protein levels in each sample. Equivalent protein amounts were separated by SDS-PAGE and transferred to PVDF membranes (Millipore). These blots were then blocked for 2 h with 5% non-fat milk, followed by 4 °C incubation overnight with appropriate primary antibodies. After being washed, blots were then incubated with an HRP-conjugated antibody (1:5,000; ab205718; Abcam), after which protein levels were visualized using an Immobilon Western Chemiluminescent HRP Substrate kit (Millipore). Primary antibodies were: rabbit polyclonal anti-EGFR (1:1000, ab5644, Abcam), anti-PTEN (1:1000, ab32199, Abcam), anti-Akt (#9272, Cell Signaling Technology, USA), anti-phospho (Ser473)-Akt (1:2000, #4060, Cell Signaling Technology), anti-MMP7 (1:1000, ab5706, Abcam), anti-MMP9 (1:1000, ab38898, Abcam), anti-E-Cadherin (1:1000, ab1416, Abcam), anti-N-Cadherin (1:1000, ab18203, Abcam), anti-Vimentin (1:1000, ab92547, Abcam).

### Pulmonary metastasis model

The role of lncZEB1-AS1 in tumor cell metastasis was assessed using a model of pulmonary metastasis. Briefly, male 6-week-old mice were randomized into 4 groups (n=5 each), and appropriate HCC cells were then intravenously injected via the tail vein. Metastases were evaluated in live mice at appropriate time points with an IVIS Lumina II system (Caliper Life Sciences, MA, USA) by injecting animals with 4 mg luciferin (Gold Biotech) in a 50 µL saline volume and then imaging animals after 10 minutes. Following a 6 week period, all mice were sacrificed, and hematoxylin and eosin (H&E) staining was used to visualize lung metastases directly. Animals were housed under standard conditions, and the guidelines of the Second Military Medical University Animal Care Facility and the National Institutes of Health were followed for all animal studies described herein.

### Statistical analysis

Data are means ± SD. Associations between lncZEB1-AS1 expression and patient clinicopathological findings were analyzed via chi-squared tests, while other data were compared via Student's t-tests or one-way ANOVAs with Tukey's post hoc test as appropriate. HCC patient survival was evaluated through a Kaplan-Meier analysis, with a log-rank test being used to gauge significance. Correlations between gene expression patterns in HCC samples were assessed via Spearman's correlation analyses. P < 0.05 was the significance threshold.

## Results

### HCC is associated with significant lncZEB1-AS1 upregulation

**Figure 1:**
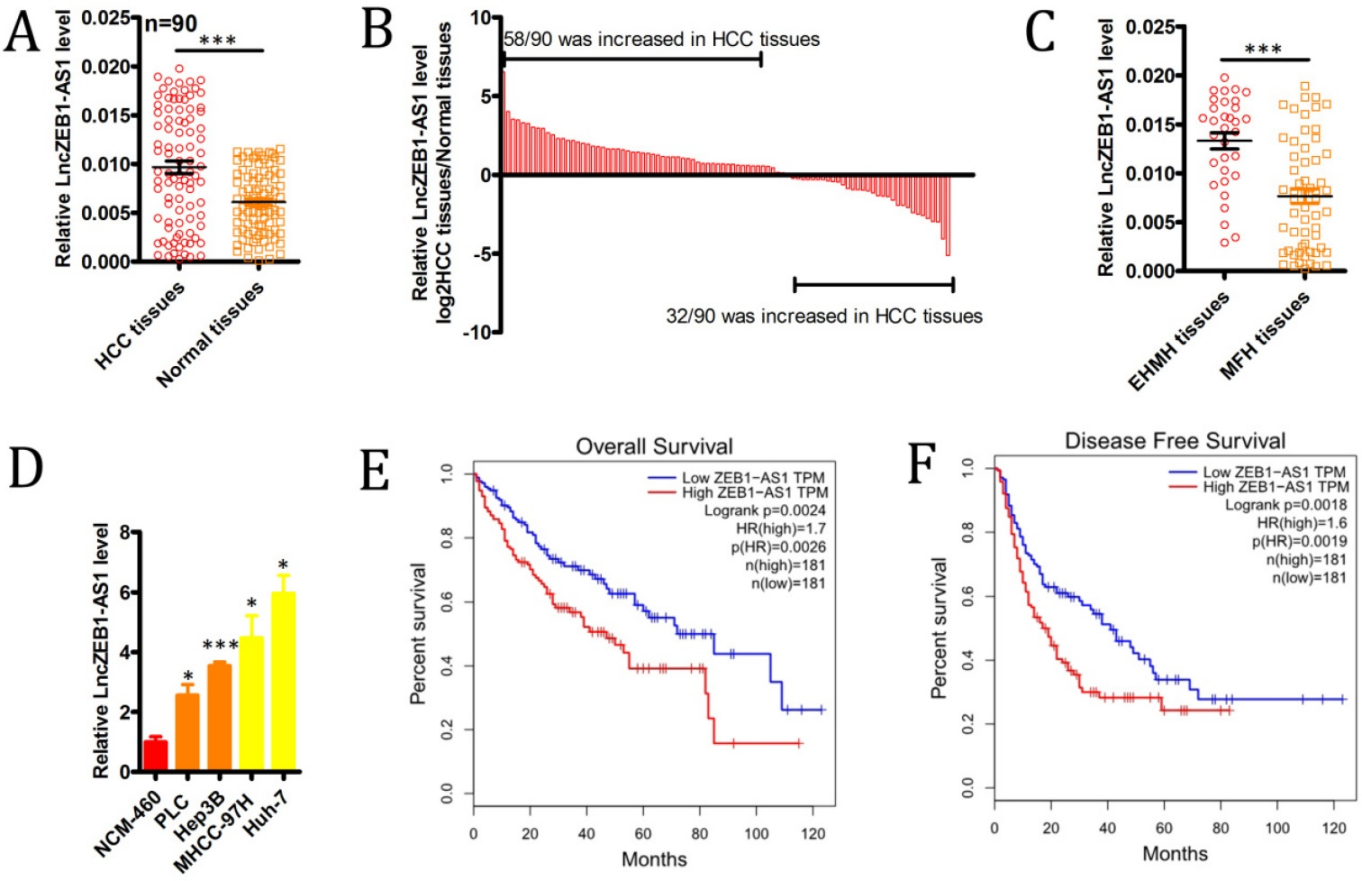
** The upregulation of lncZEB1-AS1 is associated with HCC BM and poor patient prognosis.** (**A and B**) LncZEB1-AS1 expression was assessed via qRT-PCR in 90 pairs of HCC tumor samples and ANTs. **C,** LncZEB1-AS1 expression in patients with MFH (n = 58) and EHMH (n =32) tissues. (**D**) LncZEB1-AS1 levels were compared via qRT-PCR in control or HCC (MHCC-97H, Huh-7, PLC, Hep3B) human cell lines. Data for A-D are means ± SEM and were compared via Student's t-tests. (**E and F**) Correlations between lncZEB1-AS1 expression and HCC patient overall and disease-free survival were compared via a two-sided log-rank test using data from the GEPIA database. *p < 0.05; **p < 0.01; ***p < 0.001.

We began by evaluating levels of lncZEB1-AS1 expression in 90 HCC patient tumor tissues and paired ANTs via qRT-PCR, revealing significant upregulation of this lncRNA specifically within tumor samples (Figure 1A), with this upregulation being significant in 72/90 (80.0%) samples (Figure 1B). We also determined that lncZEB1-AS1 expression levels were significantly higher in HCC patients with extrahepatic metastases (EHMH; n = 32) relative to those with metastasis-free HCC (MFH; n = 58) (Figure 1C). Consistent with these findings, we determined that the MHCC-97H and Huh-7 HCC cell lines expressed higher levels of lncZEB1-AS1 relative to control liver cell lines, and were more invasive and metastatic 26 (Figure 1D). These data confirmed that lncZEB1-AS1 upregulation was thus closely linked to HCC metastasis.

### Upregulation of lncZEB1-AS1 is closely linked to BM and poorer outcomes in HCC patients

We next explored the relevance of lncZEB1-AS1 to HCC patient metastatic progression and survival outcomes. To that end, we separated the abovementioned 90 HCC patients into lncZEB1-AS1-high and -low groups according to median levels of this lncRNA. This analysis revealed that high lncZEB1-AS1 expression was significantly associated with microvascular invasion, poor differentiation, and a lack of encapsulation (Table 1). In a univariate analysis, we found tumor encapsulation, vascular invasion, BCLC stage, and lncZEB1-AS1 levels were all associated with BM in this patient cohort (Table 2). A subsequent multivariate analysis indicated that vascular invasion, BCLC stage, and lncZEB1-AS1 expression were all independently associated with the risk of BM in HCC patients (Table 2). We then confirmed this relationship using an independent TCGA dataset of 362 HCC patients that was analyzed with the GEPIA program, confirming that higher lncZEB1-AS1 expression was significantly linked to poorer overall survival (n =362, hazard ratio (HR) = 1.7, P (HR) = 0.0026, log-rank P = 0.0024) and disease-free survival (n = 362, HR = 1.6, P (HR) = 0.0019, log-rank P = 0.0018) (Figure 1E and F). These results thus suggested that lncZEB1-AS1 may be an important regulator of BM in HCC patients.

### LncZEB1-AS1 knockdown impairs the growth and metastasis of HCC cells

**Figure 2:**
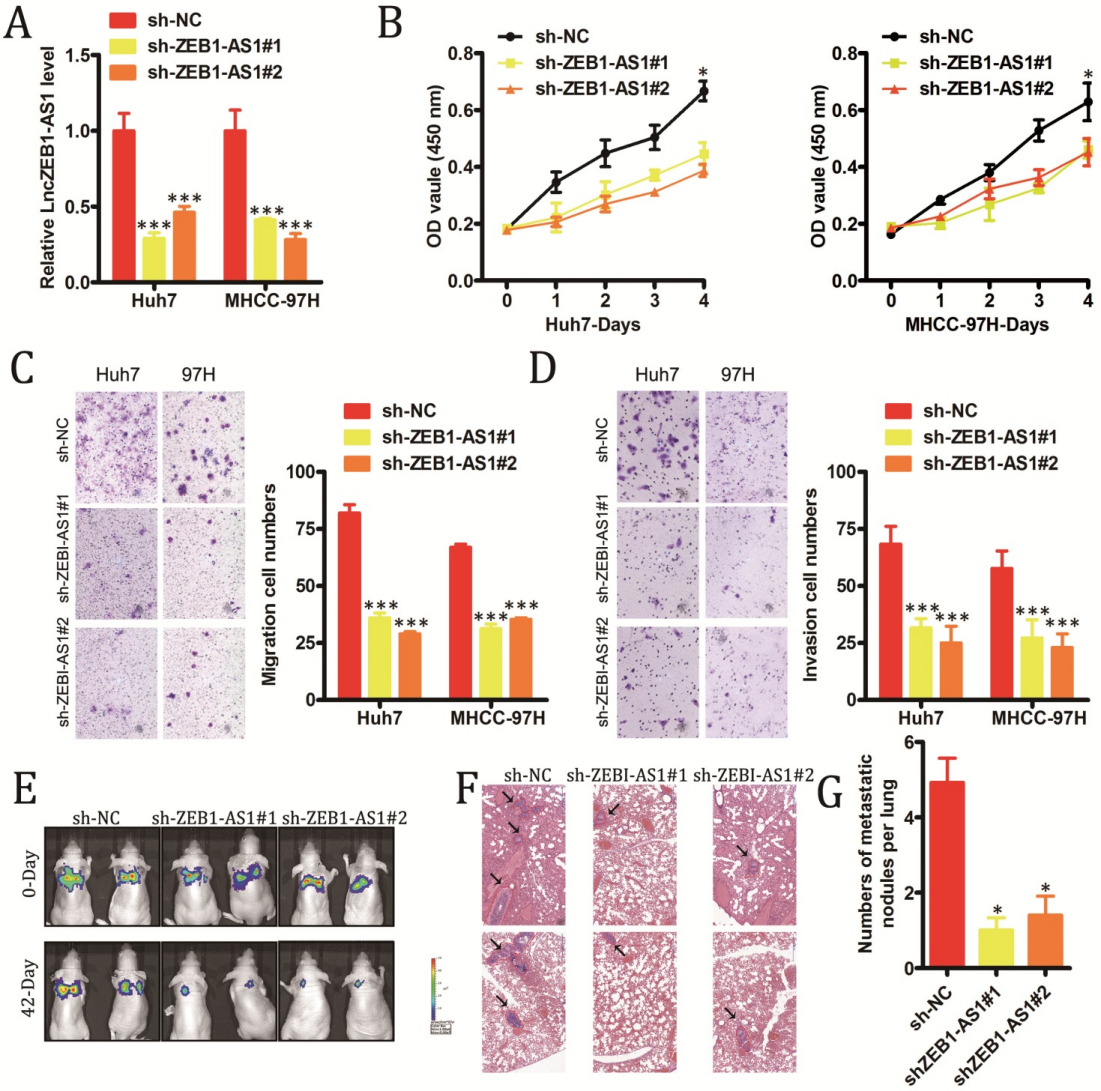
** LncZEB1-AS1 promotes HCC cell growth and metastasis.** (**A**) Confirmation of successful lncZEB1-AS1 knockdown (KD, shLncZEB1-AS1#1, 2) in Huh-7 and MHCC-97H cells. (**B-D**) The impact of lncZEB1-AS1 knockdown on HCC cell proliferation (B). migration (C) and invasion (D). (**E**) Lung metastases in mice intravenously injected with HCC cells were visualized using an IVIS Imaging System on days 0 and 48, with quantified data shown in (**F**). (**G**) Representative H&E-stained lung metastases, with quantification shown in (**H**). Scale bar, 50 mm. Data are means ± SEM, and were analyzed via Student's t-tests or repeated measures ANOVAs as appropriate. *p < 0.05; **p < 0.01; ***p < 0.001.

In order to explore the functional roles of lncZEB1-AS1 in HCC, we next modulated the expression of this lncRNA *in vitro* in HCC cells. We utilized specific shRNA constructs to knock down lncZEB1-AS1 in Huh7 and MHCC-97H cells, confirming successful knockdown via qRT-PCR (Figure 2A). Knockdown of this lncRNA was shown to markedly impair the proliferation of these HCC cells in a CCK-8 assay (Figure 2B), and to suppress tumor cell invasion and migration in a Transwell assay system (Figure 2C and D). To explore the *in vivo* relevance of these findings, we next utilized a murine model of pulmonary metastasis in which mice were injected with specific HCC cell lines. Metastatic nodules in the lungs of these mice were imaged every 2-3 weeks, revealing that these metastases grew much more slowly in animals injected with HCC cells in which lncZEB1-AS1 had been knocked down (Figure 2E and F). Following a 6-week period, these animals were sacrificed, and H&E staining of collected lungs confirmed that the knockdown of this lncRNA in HCC cells was associated with significantly reduced micro-metastasis formation in these animals (Figure 2G and H). Together, these results thus provide strong evidence that lncZEB1-AS1 facilitates the development and/or progression of HCC.

### LncZEB1-AS1 promotes PI3K-AKT signaling in order to induce MMP-2, -7 and -9 upregulation

**Figure 3:**
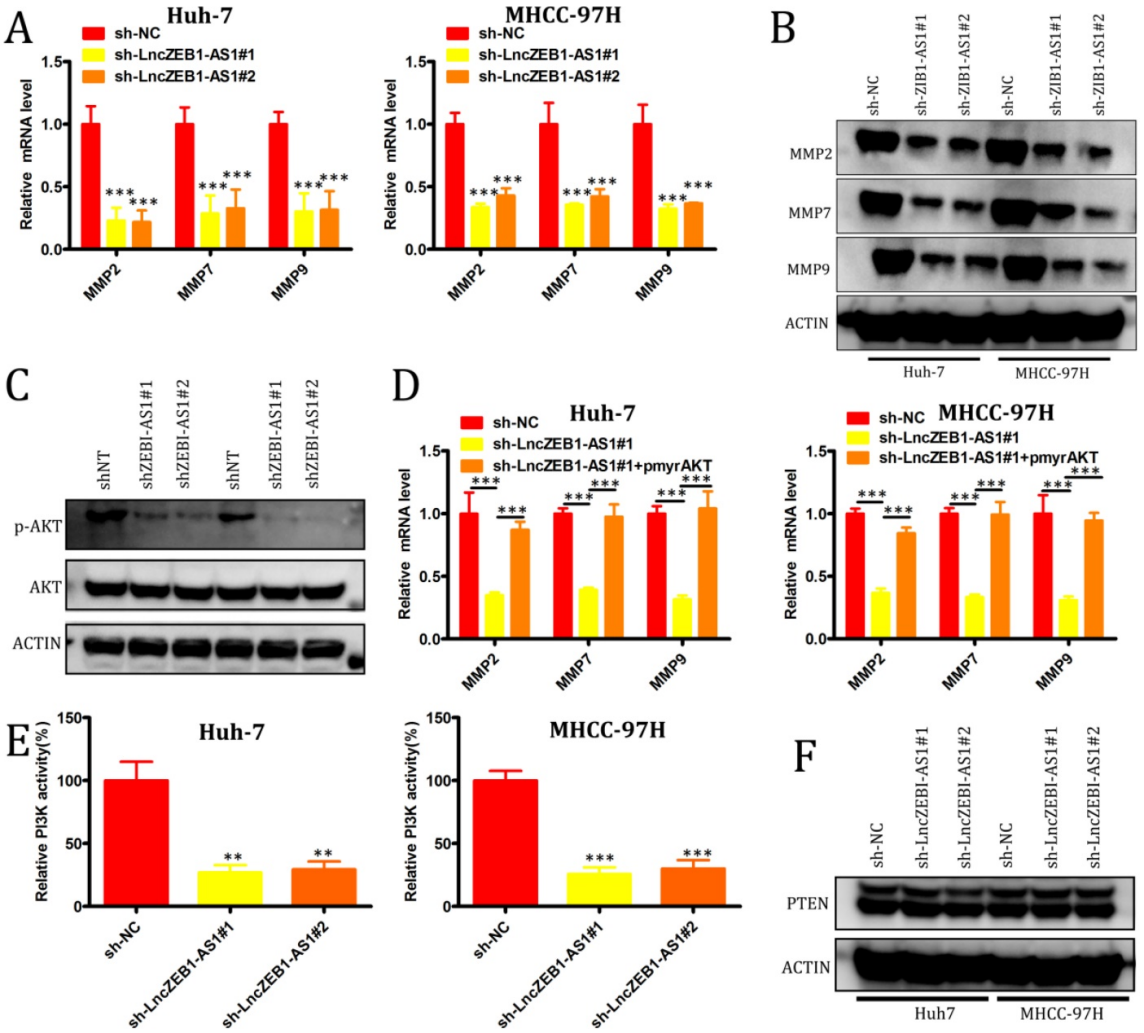
** LncZEB1-AS1 controls MMP-2, -7, and -9 expression via the activation of PI3K-AKT signaling.** (**A and B**) qRT-PCR and Western blotting were used to quantify MMP-2, -7, and -9 levels in HCC cells. (**C**) Total AKT expression and AKT phosphorylation (p-AKT) were assessed in the indicated cells via Western blotting. (**D**) Relative MMP-2, MMP-7, and MMP-9 expression in cells treated as above was assessed via qRT-PCR. (**E**) PI3K activity was assessed in the indicated cells. (**F**) PTEN expression was assessed by Western blotting in the indicated cells. GAPDH expression was used for normalization in all experiments. Data are means ± SEM, and were compared via Student's t-tests. *p < 0.05; **p < 0.01; ***p < 0.001.

We next evaluated the role of lncZEB1-AS1 as a regulator of the epithelial-mesenchymal transition (EMT), in HCC cells, given that this transition is a key step in tumor cell oncogenic progression. No effect of lncZEB1-AS1 knockdown on the expression of the EMT-associated genes vimentin, N-cadherin, or E-cadherin was observed (Supplemental Figure 1A and B), suggesting that this lncRNA does not directly impact EMT progression. We therefore next assessed the relationship between lncZEB1-AS1 and the expression of matrix metalloproteinases (MMPs), given that they are essential mediators of tumor-driven proteolysis of the extracellular matrix and subsequent invasion. We found that knockdown of lncZEB1-AS1 was associated with a significant reduction in the mRNA level expression MMP2, MMP7, and MMP9 (Figure 3A and B), and with a marked decrease in AKT phosphorylation (Figure 3C) in both tested HCC cell lines. When we ectopically expressed pmyr-AKT in cells in which lncZEB1-AS1 had been knocked down, this was associated with significant upregulation of MMP2, MMP7, and MMP9 at the mRNA level (Figure 3D). When we additionally conducted kinase activity assays and Western blotting, we were able to confirm that PI3K activity was regulated by lncZEB1-AS1, whereas this lncRNA had no comparable effect on phosphatase and tensin homolog deleted on chromosome 10 (PTEN) (Figure 3E and F). These results thus suggest that lncZEB1-AS1 functions as a promoter of HCC metastasis at least in part through a PI3K-AKT-dependent mechanism associated with the enhanced upregulation of proteolytic MMP enzymes.

### LncZEB1-AS1 targets miR-302b in order to upregulate EGFR and thereby activate PI3K-AKT signaling

**Figure 4:**
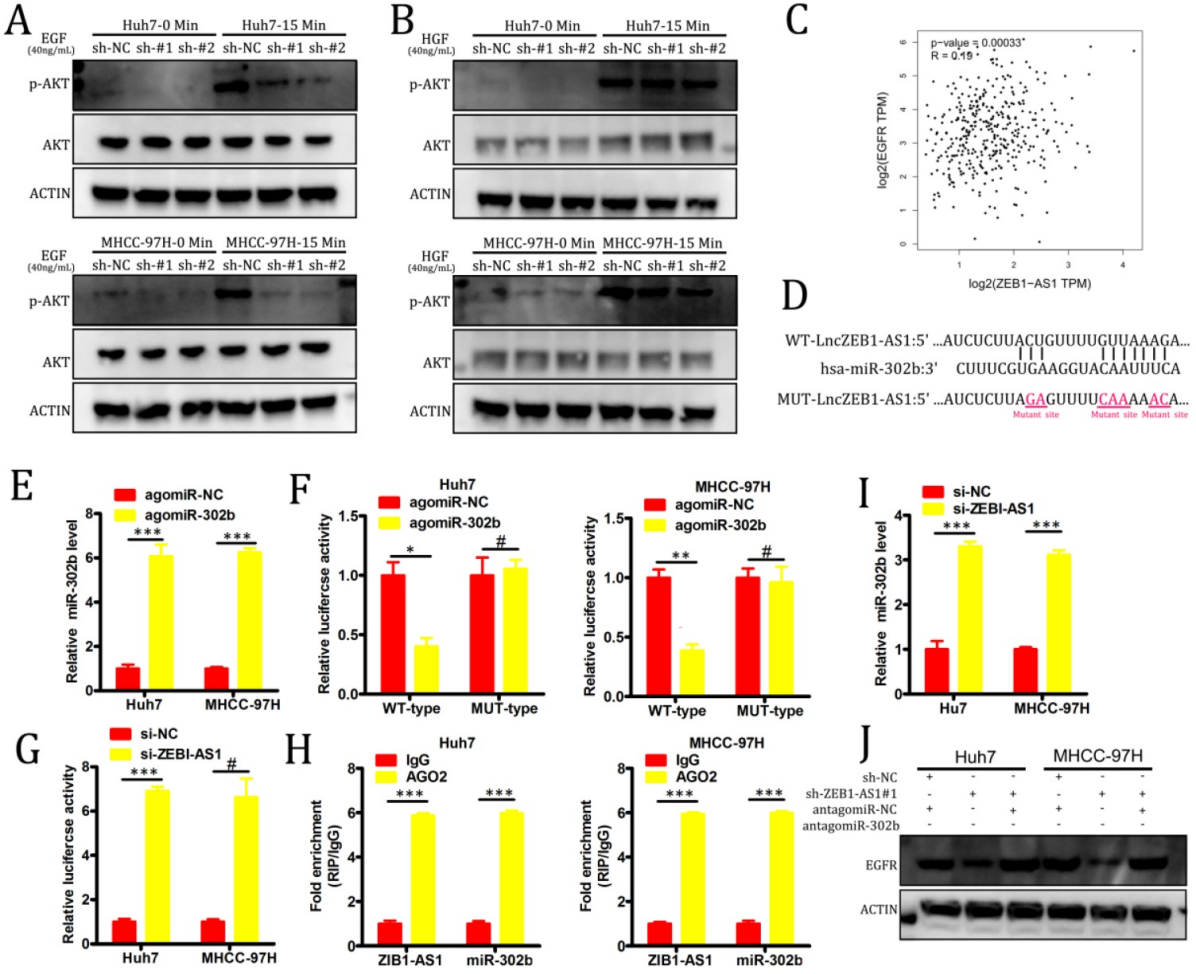
** LncZEB1-AS1 targets miR-302b in order to upregulate EGFR and to thereby promote PI3K-AKT signaling.** (**A and B**) Cells were treated using EGF (A) or HGF (B) for 0 - 15 minutes, after which Western blotting was conducted in order to quantify the expression of the indicated proteins. GAPDH served as a loading control. (**C**) A positive correlation between EGFR and lncZEB1-AS1 expression was detected in the GEPIA dataset via Pearson's correlation test. (**D**) Bioinformatic analyses predicted an interaction between miRNA-302b and lncZEB1-AS1. (E) The expression of miRNA-302b in Huh7 and MHCC-97H cells was assessed following agomir-302b or agomir-NC transfection. (**F**) Wild type (WT)-lncZEB1-AS1 or mutant (MUT)-lncZEB1-AS1 were co-transfected into HCC cells together with agomir-302b p or agomir-NC. After 48 h, luciferase activity was assessed. (**G**) Increased ZEB1-AS1 and miR-302b levels were evident in Ago2-containing immunoprecipitates relative to precipitates prepared using a control IgG. (**I**) qRT-PCR was used to assess miR-302b expression in HCC cells following antagomir-302b or antagomir-NC transfection. (**J, K**) EGFR expression was assessed via qRT-PCR and Western blotting following the co-transfection of HCC cells with si-ZEB1-AS and antagomir-302b or antagomir-NC. *P < 0.05; **P < 0.01; ***P < 0.001; #P>0.05

PI3K-AKT signaling pathway activation is primarily induced as a result of signaling through specific receptor tyrosine kinases (RTKs) in response to specific ligand binding events. In order to evaluate the role of RTKs in our experimental system, we treated our HCC cell lines with either hepatocyte growth factor (HGF) or EGF. We found that the knockdown of lncZEB1-AS1 markedly impaired EGF-induced AKT activation in both Huh7 and MHCC-97H cells, whereas no such changes were observed in response to HGF stimulation (Figure 4A-B). In a GEPIA analysis, we additionally determined that there was a significant positive correlation between the expression of EGFR and lncZEB-AS1 in HCC patient tumor samples (Figure 4C), suggesting that this lncRNA may promote HCC progression via an EGFR-PI3K-AKT axis. In many cases, lncRNAs have been shown to act as competing endogenous RNAs (ceRNAs) capable of binding to specific miRNAs and thereby interfering with their ability to suppress target gene expression 17. Using starBase 3.0, we were able to identify miR-302b as a putative binding target for lncZEB1-AS based on sequence complementarity (Figure 4D). Given that this miRNA has also been found to function as a tumor suppressor in the context of HCC progression through its ability to target EGFR 27, we selected it for further validation. Using a luciferase reporter assay system, we were able to confirm that a WT but not a MUT version of lncZEB1-AS1 was able to bind to agomiR-302b such that this miRNA only altered luciferase activity when transfected into cells that had been co-transfected with a WT-lncZEB1-AS1 reporter construct (Figure 4E-F). We were further able to confirm a direct interaction between lncZEB1-AS1 and miR-302b via a RIP assay which confirmed the significant enrichment of both of these RNAs in Ago2-containing immunoprecipitates but not in precipitates prepared using control IgG (Figure 4G). When lncZEB1-AS1 was knocked down in Huh7 and MHCC-97H cells, this was associated with a significant increase in miR-302b expression in these same cells (Figure 4H). These results therefore supported a model wherein lncZEB1-AS1 functions as a ceRNA for miR-302b in HCC cells. We next assessed whether lncZEB1-AS1 was able to enhance EGFR expression in HCC cells via miR-302b sequestration by knocking down this lncRNA in cells that had been transduced with either antagomiR-302b or a control antagomir construct. We initially confirmed that antagomiR-302b transfection significantly reduced miR-302b levels in both Huh7 and MHCC-97H cells (Figure 4I), after which we confirmed that antagomiR-302b transfection was sufficient to reverse mRNA (Figure 4J) and protein (Figure 4K) levels decreased in EGFR expression in cells in which lncZEB1-AS1 had been knocked down. Together, these findings thus indicated that lncZEB1 functions as a positive regulator of EGFR expression in HCC cells at least in part owing to its ability to function as a “molecular sponge” for miR-302b.

## Discussion

Advanced solid tumors including those of the lung, breast, and prostate often metastasize to the bone 28, 29, with BM being associated with an extremely poor patient prognosis 30. It is vital that the molecular mechanisms governing HCC BM be better studied, as at present they are not well understood. In previous studies, elevated lncZEB1-AS1 expression has been detected in HCC tumor samples relative to ANTs, and this lncRNA has been shown to be correlated to tumor growth and metastasis in HCC patients 18, 24. The specific role of lncZEB1-AS in HCC BM and any underlying molecular mechanisms, however, have not been previously assessed. In this study, we therefore examined the role of lncZEB1-AS1 in HCC development and metastatic progression.

Initially, we confirmed that lncZEB1-AS1 was upregulated in HCC tumor tissue samples relative to healthy adjacent tissue, and we confirmed that there was a positive correlation between the expression of this lncRNA and the incidence of BM in HCC patients. We next examined the molecular mechanisms whereby lncZEB1-AS1 impacts HCC metastatic progression. While EMT is a key process related to tumor cell metastasis 31-34, we found that knocking down lncZEB1-AS1 did not alter EMT-related protein expression in HCC cell lines. However, we did find that changes in lncZEB1-AS1 expression were closely linked to changes in AKT activation and associated signaling in these cells. Through kinase activity assays and qRT-PCR, we ultimately found that lncZEB1-AS1 can enhance PI3K-AKT signaling in order to upregulate MMP2, MMP7, and MMP9 in HCC cells, thereby promoting tumor metastasis, consistent with prior reports highlighting the importance of AKT signaling in HCC onset and progression 35-41. Our data further suggested that lncZEB1-AS1 promotes EGFR upregulation in HC cells, thereby enhanced EGF- but not HGF-mediated activation of PI3K-AKT signaling. While lncRNAs can alter tumor progression through several different mechanisms, the best understood such mechanism is that in which lncRNAS serve as ceRNAs for specific target miRNAs 42. In prior studies, lncZEB1-AS1 has been shown to serve as a ceRNA for miR-133b 43, miR-942 44, miR-455-3p 45, miR-141-3p 30, miR-409-3p 46, miR-1224-5p 47, and miR-181a-5p 48 in a range of tumor types. Through predictive analyses, we identified miR-302b as a novel lncZEB1-AS1 target miRNA that has also previously been shown to function as a tumor suppressor in HCC owing to its ability to target EFGR 27. Through luciferase reporter and RIP assays we were able to confirm that lncZEB1-AS1 can directly interact with miR-302b in HCC cells, and we were additionally able to confirm that lncZEB1-AS1 knockdown significantly increased miR-302b expression in these HCC cells, thereby resulting in EGFR downregulation. Together, our results therefore support a model in which lncZEB1-AS1 functions as a ceRNA for miR-302b in order to indirectly regulate EGFR expression in HCC cells.

EGFR signaling is known to be associated with many different solid tumor types including colon cancer, non-small cell lung cancer (NSCLC), breast cancer, renal cancer, head and neck cancer, and glioma 49-51. In NSCLC, for example, EGFR overexpression is correlated with patient histopathological findings, and with the degree of tumor aggression and invasion. As a result, many studies have sought to interfere with EGFR signaling pathways in tumors in which this signaling pathway is dysregulated, resulting in beneficial clinical outcomes in multiple different solid tumor types 52-54. Although further research is needed in order to extend these findings to HCC, there is increasingly robust preclinical evidence suggesting that EGFR-targeting agents may be effective in the treatment or prevention of HCC 55, 56. Our findings further suggest that compounds targeting the lncZEB1-AS1‐miR-302b-EGFR axis may be valuable as a means of preventing BM in HCC patients.

In summary, we have provided novel evidence suggesting that lncZEB1-AS1 is an important regulator of HCC BM. We have additionally highlighted a previously undescribed lncZEB1-AS1-miR-302b-EGFR-PI3K-AKT signaling axis that may govern this metastatic process. This axis may be a viable target for therapeutic intervention in HCC patients as a means of significantly improving quality of life and extending patient survival.

## Supplementary Material

Supplementary figure S1.

## Figures and Tables

**Table 1:** Correlation of clinic-pathologic characteristics with LncZEB1-AS1 level

Characteristics	ZEB1-AS1 level		P value
	High (n = 45)	Low (n = 45)	
**Age, y**			0.399
≤50	24	20	
>50	21	25	
**Gender**			0.777
Male	37	38	
Female	8	7	
**Liver cirrhosis**			0.378
Yes	27	31	
No	18	14	
**AFP, ng/mL**			0.284
≤20	16	21	
>20	29	24	
**Tumor differentiation**			0.011*
I-II	5	15	
III-IV	40	30	
**Tumor size, cm**			0.266
≤5	26	29	
>5	19	16	
**Tumor number**			0.517
Single	36	38	
Multiple	9	7	
**Microvascular invasion**			0.030*
Yes	29	38	
No	16	7	
**Pathological satellite**			0.694
Yes	41	42	
No	4	3	
**Tumor encapsulation**			0.038*
Complete	27	35	
None	18	10	
**Hepatitis B virus DNA**			0.527
>1.0e + 03 IU/mL	23	20	
<1.0e + 03 IU/mL	22	25	
**BCLC stage**			0.581
0-A	36	38	
B	9	7	
**TNM stage**			0.520
I	25	28	
II + III	20	17	

Note: The χ2 test was used for comparison between groups. Abbreviations: AFP, α-fetoprotein; BCLC, Barcelona Clinic Liver Cancer staging; *P <0.05.

**Table 2:** Univariate and multivariate analyses of factors associated with bone metastasis in 90 HCC patients

Variable Bone metastasis	Univariate analyses			Multivariate analyses		
	HR	95% CI	P	HR	95% CI	P
Age > 50 versus ≤50 years	1.867	0.720-2.304	0.107	-	-	-
Gender male versus female	0.702	0.629-1.866	0.968	-	-	-
Liver cirrhosis Yes versus No	1.771	0.733-2.625	0.551	-	-	-
AFP, ng/mL >20 versus ≤20	1.463	0.419-2.829	0.114	-	-	-
Tumor differentiation III-IV versus I-II	1.892	0.563-3.317	0.631	-	-	-
Tumor size, cm > 5 versus ≤ 5	0.684	0.458-1.534	0.345	-	-	-
Tumor number multiple versus single	1.398	0.582-1.964	0.406	-	-	-
Vascular invasion Yes versus No	2.492	1.312-3.649	0.031	2.112	1.314-3.162	0.034
Pathological satellite Yes versus No	1.323	0.739-3.962	0.629	-	-	-
Tumor encapsulation complete versus none	2.625	1.739-4.404	0.022	1.892	0.537-2.461	0.234
Hepatitis B virus DNA, IU/mL >1.0e + 03 versus <1.0e + 03	1.367	0.791-2.169	0.932	-	-	-
BCLC stage B versus 0-A	1.702	1.371-3.194	0.019	1.526	1.712-2.622	0.045
TNM stage II + III versus I	1.524	0.394-2.417	0.338	-	-	-
LncZEB1-AS1 high versus low	2.767	1.460-2.061	0.009	2.278	1.240-3.507	0.015
