# Supplementary material for: LncZEB1-AS1 regulates hepatocellular carcinoma bone metastasis via regulation of the miR-302b-EGFR-PI3K-AKT axis
Source: J Cancer. 2020 Jun 28;11(17):5118–28. doi: 10.7150/jca.45995 (PMC7378930; doi:10.7150/jca.45995)

LncZEB1-AS1 mediated regulation of bone metastasis in hepatocellular carcinoma by miR-302b-EGFR-PI3K-AKT aixs

Zhen-jiang Ma[1, 2 *], Yao Wang[3*], Hui-fen Li[4*], Ming-Hua Liu[3], Feng-rui Bi[3], Long Ma[3], Hui Ma[1#], Hong-li Yan[3#]

1. Department of Orthopedics, the Third Affiliated Hospital of Second Military Medical University, Shanghai 201805, P.R. China;
2. Department of Orthopedics, Shanghai Ninth People's Hospital, Shanghai 200011, P.R. China
3. Department of Laboratory Medicine, Changhai Hospital, Second Military Medical University, Shanghai, P.R. China
4. Department of Interventional, Eastern Hepatobiliary Surgery Hospital, Second Military Medical University, Shanghai, P.R. China

* Contribution equality

Correspondence: Hui Ma, Department of Orthopedics, the Third Affiliated Hospital of Second Military Medical University, Shanghai 201805, P.R. China; Email: huima@smmu.edu.cn; Hong-li Yan, Department of Laboratory Medicine, Changhai Hospital, Second Military Medical University, Shanghai, P.R. China; Email: hongliyan@smmu.edu.cn.

Supplement-Figure S1:

A, The mRNA and protein expressions of vimentin, E-cadherin and N-cadherin between the specified cell lysates were compared by qRT-PCR and Western blot analysis.* P<0.05; ** P<0.01; *** P<0.001; #P>0.05.

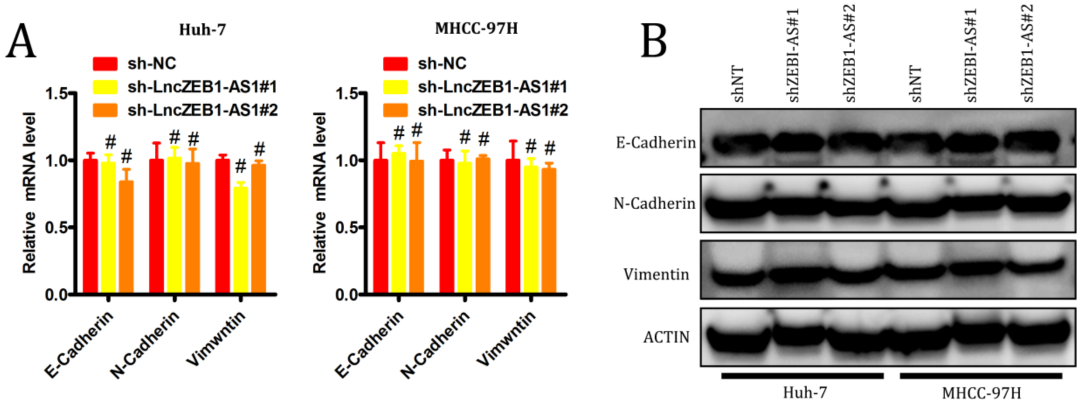

Supplement: Supplementary file 1 — Supplementary figure S1. [file jcav11p5118s1.pdf]
